# Seroprevalence, risk factors, and spatial distribution of epizootic hemorrhagic disease virus in dromedary camels from northern Oman

**DOI:** 10.14202/vetworld.2026.877-887

**Published:** 2026-02-28

**Authors:** Muhammad Hammad Hussain, Elshafie Ibrahim Elshafie, Haytham Ali, Khalid Al Habsi, Al Ghalya Al Toubi, Abeer Al-Hamrashdi, Mazen Al-Harrasi

**Affiliations:** Department of Animal and Veterinary Sciences, College of Agricultural and Marine Sciences, Sultan Qaboos University, Al Khoudh, Muscat 123, Oman

**Keywords:** dromedary camels, EHDV antibodies, epizootic hemorrhagic disease, northern Oman, risk factors EHDV, seroprevalence study, spatial clustering EHDV

## Abstract

**Background and Aim:**

Epizootic hemorrhagic disease virus (EHDV), an *Orbivirus* transmitted by *Culicoides* midges, infects a wide range of ruminant species including dromedary camels (*Camelus dromedarius*). Although circulation of EHDV has been documented in cattle in the Sultanate of Oman, its epidemiology in camels remains poorly characterized. This study was conducted in the Al Batinah North and Al Batinah South governorates, coastal regions with hot, arid climates favorable to vector activity, to estimate individual-level seroprevalence of EHDV antibodies in dromedary camels, identify associated risk factors using univariate and multivariate analyses, and evaluate spatial distribution patterns to inform future control measures in multi-host livestock systems.

**Materials and Methods:**

A cross-sectional study was performed between September 2020 and July 2021. Serum samples were collected from 415 camels across 50 holdings using convenience sampling. Antibodies against EHDV were detected with a commercial competitive enzyme-linked immunosorbent assay (c-ELISA) targeting the VP7 protein (sensitivity = 85.9 %, specificity = 99.7 %). True prevalence (TP) was calculated using the Rogan–Gladen estimator. Univariate associations were assessed with chi-square or Fisher’s exact tests; variables with p < 0.25 were entered into backward stepwise binary logistic regression. Spatial analyses included average nearest-neighbour clustering, Getis-Ord Gi* hot-spot analysis, and kernel density estimation in ArcGIS.

**Results:**

Apparent seroprevalence was 40.2 % (95 % confidence interval [CI]: 35.5–45.1 %), with an estimated TP of 46.7 % (95 % CI: 41.3–52.3 %). No significant difference existed between governorates (*p* = 0.520). In univariate analysis, seropositivity was significantly higher in camels > 4 years of age (45.9 % vs 19.3 %; p < 0.001), in breeding/leisure camels (43.1 % vs 20.8 %; p = 0.002), and in animals kept in contact with other ruminants (49.3 % vs 35.6 %; p = 0.007). After multivariate adjustment, only age > 4 years (odds ratio [OR] = 3.4, 95 % CI: 1.88–5.96; p < 0.01) and contact with other ruminants (OR = 1.6, 95 % CI: 1.05–2.44; p = 0.029) remained independent risk factors. Global spatial clustering of positive holdings was highly significant (nearest-neighbour ratio = 0.39, z = –8.26, p < 0.001), with elevated density along coastal agricultural corridors.

**Conclusion:**

This first large-scale serological survey confirms active circulation of EHDV among dromedary camels in northern Oman. Older age and mixed-species herding are the main risk factors identified. The findings highlight the importance of camels in the multi-host ecology of EHDV and emphasize the need for integrated national surveillance, vector studies, and molecular characterization of circulating serotypes to support evidence-based control strategies in a changing climate.

## INTRODUCTION

The epizootic hemorrhagic disease virus (EHDV) is an *Orbivirus* that can infect multiple ruminant species, including camels [1]. The virus is closely related to Bluetongue virus (BTV) and shares the vectors (*Culicoides* spp.), climatic (temperate and tropical) regions, and geographical distribution with BTV [2]. The clinical manifestations of EHDV infection can vary greatly depending on the host species, individual animal, and specific viral serotype or strain [3]. There are seven recognized EHDV serotypes (EHDV-1, 2, 4, 5, 6, 7, and 8) with two recently suggested additional new putative serotypes [4, 5]. The Ibaraki virus, which is associated with severe disease in Japanese cattle, is currently reclassified as EHDV serotype 2 [2].

The geographic distribution of EHDV is linked to the distribution of *Culicoides* vectors found in diverse temperate and tropical regions worldwide [6, 7]. Generally, warm temperatures and high humidity are considered to be the optimal climatic conditions for robust vector activity [2, 8]. Warmer latitudes or climates favor the year-round activity of midges and thus EHDV circulation in susceptible species [6, 8]. Higher temperatures further facilitate transmission by reducing the extrinsic incubation period of the viruses within the midges; for instance, infection rates are markedly elevated above 20°C compared to 15°C [2, 9, 10]. Environments abundant in organic matter, such as accumulated animal feces near water sources, along with wetlands featuring grassland, are also conducive to the reproduction and feeding of *Culicoides* [8, 10]. Global climate change is projected to expand the geographical range and seasonal abundance of *Culicoides* vectors, increasing the risk of EHDV outbreaks in previously disease-free areas [2, 11].

The most devastating clinical impact of EHDV is seen on the deer as a hemorrhagic disease [1] with high morbidity and mortality rates as high as 90% [12, 13]. The clinical disease in deer could vary from acute to chronic. In cattle, EHDV infection could resemble BTV infection and cause serious economic losses to the dairy industry through lowered milk production and abortions [3, 14]. Clinical infection and signs in yaks have also been reported with production losses and abortions [15]. Although clinical disease is seldom reported in goats and sheep [16, 17], the presence of antibodies and viral nucleic acid has been frequently reported in these animals [14, 18, 19]. Furthermore, occasional mortality due to EHDV has been reported in bison and elk herds in the USA [12]. Serological evidence of EHDV infection has also been reported in other wild ruminants, including mountain goats, ibex, rhinoceros, bears, and marsupials [2].

Despite increasing reports of EHDV circulation among ruminants in the Middle East, North Africa, and South Asia, important gaps remain in understanding its epidemiology in dromedary camels (*Camelus dromedarius*). Although EHDV antibodies have been detected in camels from the United Arab Emirates (29%), Pakistan (6.5–50%), Sudan (17%), and Mauritania (73%) [20–22], most of these studies were limited in sample size, lacked detailed risk factor assessment, or did not include spatial analyses. In the Sultanate of Oman, recent serological evidence has confirmed substantial EHDV exposure in cattle, with an apparent seroprevalence of approximately 53% in selected governorates [23]; however, no large-scale investigation has yet examined EHDV seroprevalence, associated risk factors, or spatial patterns in the country’s significant dromedary camel population. Camels in Oman are frequently managed in mixed-species systems alongside cattle, sheep, and goats, conditions that may facilitate cross-species transmission by shared *Culicoides* vectors, yet their contribution to the multi-host ecology of EHDV remains largely unexplored. This paucity of camel-specific data, particularly from northern coastal regions with hot, humid conditions conducive to vector activity, limits the ability to design evidence-based surveillance and control measures in a region increasingly influenced by climate change [2, 11].

The aim of this study was to estimate the individual-level seroprevalence of EHDV antibodies in dromedary camels from Al Batinah North and Al Batinah South governorates of northern Oman, to identify host- and management-related risk factors associated with seropositivity using univariate and multivariate statistical analyses, and to evaluate the spatial distribution of EHDV-positive holdings to provide baseline information and inform future integrated surveillance and control strategies within multi-host livestock systems in the region.

## MATERIALS AND METHODS

### Ethics approval and consent to participate

This study involved non-invasive blood sampling from clinically healthy dromedary camels performed by licensed veterinarians following standard veterinary procedures. Informed verbal consent was obtained from all camel owners prior to sampling, explaining the study purpose, procedures, and voluntary nature of participation. No animals were harmed, sedated, or subjected to any experimental treatment beyond routine jugular venipuncture. The study protocol was reviewed and approved by the Ethics Committee for the Research, Sultan Qaboos University, Muscat, Oman (approval reference: SQU/EC-AUR/2025-2026/10). All procedures complied with the institutional animal welfare guidelines and the Omani regulations for the care and use of animals in research.

### Study period and location

**Figure 1:**
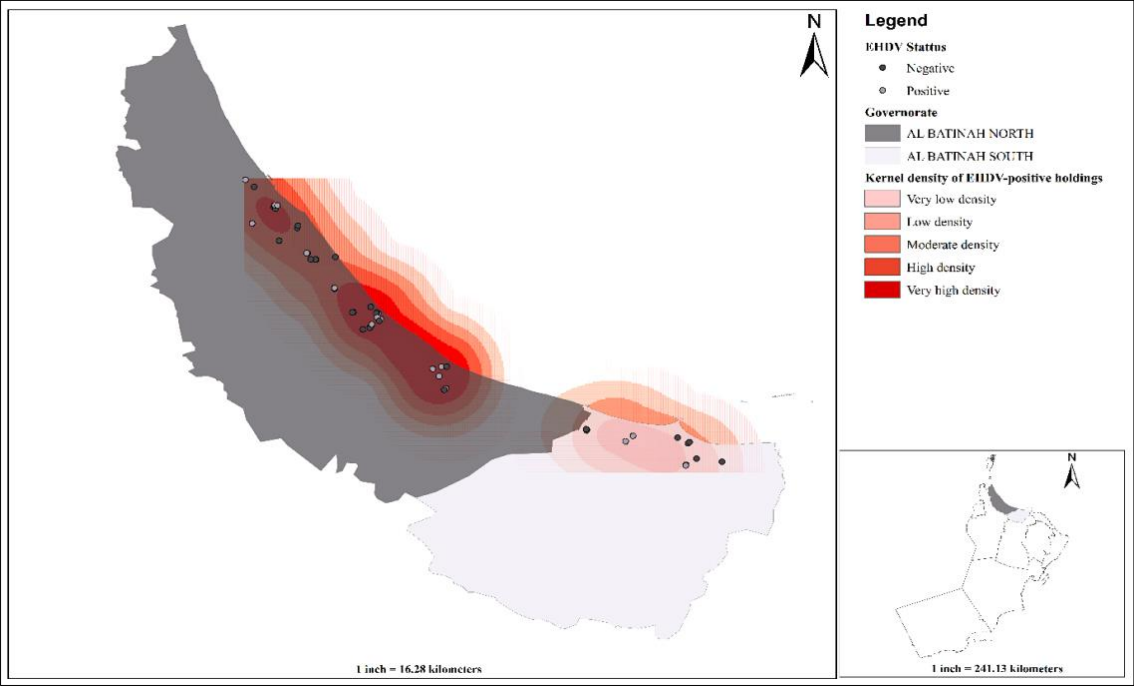
Spatial distribution and kernel density of epizootic hemorrhagic disease virus-positive camel holdings (n = 50) in northern Oman’s Al Batinah North and South governorates.

The sampling was conducted between September 2020 to July 2021 and laboratory work and data analysis was conducted between January 2024 to December 2024. The study was conducted in Al Batinah North and Al Batinah South governorates of northern Oman, located along the Batinah coastal plain (approximately 24°N, 56°–57°E) and targeted an estimated camel population of around 20,000 heads [24]. These governorates share similar climatic conditions (Figure 1), characterized by a hot, arid climate with annual mean temperatures around 28°C–29°C and limited rainfall. The summer is extremely hot and humid, whereas the winters are milder. The elevation in the sampled governorates generally ranged from approximately 0 to <200 m above sea level.

### Sampling frame and target population

The sample size was calculated for a disease of unknown prevalence (50% expected prevalence) at 95% confidence level and 5% desired absolute precision [25] using the following formula:

n = (1.96)² × P(exp) × (1 – P(exp)) / d²

Where: n is the required sample size, P(exp) is the expected prevalence, d is the desired absolute precision, and 1.96 is the multiplier associated with the 95% CI.

The minimum required sample size for the cross-sectional study was 384 camels. During the study period, 415 camels from 50 owners/locations were sampled for the estimation of individual-level seroprevalence, and no design effect was incorporated as the analysis was conducted at the animal level without additional clustering adjustment. Camel owners were chosen with the help of local veterinary clinics and camel owner associations. All herds included in the study belonged to private farmers. In general, these animals were kept in open-fenced enclosures close to permanent homes or farms, a common management method in the sampled governorates. Therefore, the study likely reflected typical practices well. Camels were identified numerically within a holding, and a lottery method was used for random selection. All sampled camels were clinically examined at the time of blood collection and were found to be clinically healthy (i.e., without showing any signs of fever, oral lesions, and lameness). The age of each camel was initially obtained from the owner’s report and subsequently verified by dental examination of the rostral dentition [26]. The presence or absence of tick infestation was also recorded on clinical examination, but no ticks were collected from the infested camels. Approximately 5-7 mL blood was aseptically drawn from the jugular veins via vacutainers (VACUETTE® TUBE 7 mL, Greiner Bio-One, Monroe, NC, USA), and epidemiological information along with geographical coordinates were recorded.

### Serological investigation

Blood samples were transported to the laboratory at approximately 4°C within 3 h of completing field collection. A USB temperature and humidity data logger (TempU03, Tzone Digital Technology Co., Ltd., Shenzhen, China) was used for cold-chain monitoring during transport. On arrival, sera were immediately separated and subsequently stored at –20°C until aliquots were obtained for serological testing. For the primary serological testing, each sample was thawed once, and for re-testing of doubtful results a separate aliquot from the second cryovials was thawed to avoid repeated freeze–thaw cycles. Serum samples were tested for EHDV antibodies using a commercial competitive enzyme-linked immunosorbent assay (ELISA) targeting the conserved VP7 core protein (ID Screen® EHDV Competition, Innovative Diagnostics, Grabels, France). Although this assay has not been formally validated in camels, the VP7 c-ELISA format is species-independent and has been successfully applied in camel serology [4, 27]. The reported specificity (Sp) and sensitivity (Se) were 99.7% and 85.9%, respectively. The test was performed by strictly following the manufacturer’s instructions. The kit-supplied positive and negative control sera were included and processed on each ELISA plate in accordance with the manufacturer’s instructions to validate the run before interpreting the sample results. The plates were read at 450 nm, and the test results were evaluated using the following sample-to-negative ratio (S/N):

S/N = (ODsample / ODNeg) × 100.

Samples with S/N values below 30% were interpreted as positive and those above 40% as negative. Those with values between 30% and 40% were considered doubtful and retested once. Based on the second result, each animal was definitively classified as seronegative (S/N ≥40%) or seropositive (S/N ≤30%), with no samples retained as doubtful in the final analysis.

### Statistical analysis

The individual-level and epidemiological data were stored in a Microsoft Excel spreadsheet. Continuous age was grouped into ordered categories and recoded into binary indicators (e.g., young vs. old) to facilitate interpretation where appropriate. Gender was treated as a binary variable and coded 0 for the reference category and 1 for the comparison category (e.g., 0 = female, 1 = male). Other categorical predictors (e.g., breed, purpose, herd type, and location) were converted into dummy variables with 0/1 coding, with one category designated as the reference group. Apparent seroprevalence (AP) was defined as the proportion of animals testing positive via ELISA. Additionally, a 95% binomial exact confidence interval (CI) was calculated. True prevalence (TP) was estimated from the apparent seroprevalence using the Rogan–Gladen estimator. The ELISA sensitivity and specificity were fixed at 85.9% and 99.7%, respectively. Univariate analysis was conducted to assess different variables and their potential association with seropositivity. The values of chi-square (χ²) or Fisher’s exact test were used to analyze the categorical variables. The respective odds ratio (OR) and 95% CIs were also calculated for each variable. Before moving forward with multivariate analysis, collinearity was checked. Multicollinearity among predictors was evaluated using the multiple linear regression model’s tolerance and variance inflation factor (VIF) statistics. Collinearity was considered potentially problematic when tolerance was 0.10–0.20 or VIF exceeded 5.0, with VIF > 10 regarded as strong evidence of serious multicollinearity. Backward stepwise modeling was used to obtain a parsimonious, clinically interpretable model while controlling for potential confounders, and ORs were interpreted as relative changes in outcome odds per unit change in each predictor. Analysis was performed by including all variables with a p-value < 0.25 in the initial model and removing non-significant (p > 0.05) predictors at each step until only significant (p < 0.05) variables were retained. The variable selection threshold of p < 0.25 was chosen as a screening criterion to avoid prematurely excluding potentially important predictors and confounders. The model fit was assessed using Nagelkerke’s R-squared and Hosmer-Lemeshow test values. Data were analyzed using IBM SPSS software (Version 23).

Spatial data were managed and analyzed using ArcMap 10.4 (Esri Inc., Redlands, CA, USA) using the WGS 84/ UTM Zone 40N projected coordinate system (EPSG:32640), based on the World Geodetic System 1984 datum and Transverse Mercator projection (central meridian 57°E, scale factor 0.9996, false easting 500,000 m, false northing 0 m). Global spatial clustering of EHDV-positive farms was evaluated using the average nearest-neighbour (ANN) statistic in ArcGIS 10.4 (Spatial Statistics Tools). Analyses were conducted on point locations of EHDV-positive farms in a projected coordinate system (UTM) using Euclidean distance. The nearest-neighbor ratio (NNR), z-score, and p-value were used to assess deviation from randomness.

## RESULTS

### Descriptive characteristics of sampled camels

A total of 415 camels (60 male and 355 female) were sampled from Al Batinah North (n = 329) and Al Batinah South (n = 86) governorates of Oman. Samples were collected from 50 camel holdings (Figure 1). The sampled camels were categorized into two age groups: those aged ≤ 4 years (n = 88) and those aged >4 years (n = 327). This cutoff was chosen because camels typically reach sexual maturity around the age of 4 years and begin their main reproductive or working life at that point. The breed-related distribution of samples was 407 locals (Batiniyyah breed) and 8 crossbred (crosses between Batiniyyah and Dhofari) camels. For this study, camels were classified into two purpose-of-use categories: (i) race camels (n = 53), which were specifically bred, trained, and managed for participation in organized camel racing events, and (ii) leisure/breeding camels (n = 362), which were kept primarily as family-owned animals for hobby, cultural activities, and herd reproduction. Most of the sampled camels were kept for breeding and leisure (n = 362), followed by those kept for race (n = 53). Tick infestation was recorded in 65.8% (n = 273) of the camels. Most of the sampled camels were managed alone (n = 275) and maintained under low-input management in open-fenced enclosures located close to wadis and agricultural farms. No specific insect control measures (e.g., insecticide application or targeted vector management) were implemented in these units, and manure accumulation within pens and around farm infrastructure was common. Feeding practices were predominantly mixed, with camels allowed to graze natural vegetation or crop residues in surrounding areas while receiving supplementary feedstuffs, reflecting typical semi-intensive camel production systems in arid and semiarid environments.

### Seroprevalence of EHDV

The overall individual-level seroprevalence of EHDV in sampled camels was 40.2% (CI 35.5–45.1), which was slightly higher in camels from Al Batinah North (41.0%, CI 35.7–46.6) when compared with those sampled from Al Batinah South (37.2%, CI 27.0–48.3), p = 0.520. The OR for testing positive for EHDV was 1.2 times higher in BN camels than in BS camels (Table 1). Based on the reported sensitivity and specificity of the c-ELISA, the TP was 46.7% (CI 41.3–52.3). Because the ELISA had imperfect sensitivity but very high specificity, the Rogan–Gladen adjustment yielded a TP that was higher than the observed apparent prevalence.

**Table 1:** Epizootic hemorrhagic disease virus seroprevalence in camels (n = 415) from Al Batinah North and South governorates of Oman.

Governorate	Wilayat (District)	Tested	Positive	Prevalence (%) (CI)
Al Batinah North	Khabourah	57	18	31.6 (19.9–45.5)
	Liwa	26	11	42.3 (23.4–63.1)
	Saham	71	31	43.7 (31.9–56)
	Sohar	175	75	42.9 (35.4–50.5)
Al Batinah South	Barka	62	20	32.3 (20.9–45.3)
	Musanaa	24	12	50 (29.1–70.9)
Total		415	167	40.2 (35.5–45.1)

Prevalence was not significantly different between the two governorates, χ² = 0.415, p = 0.520. CI = Confidence interval.

### Spatial distribution and clustering

EHDV-positive locations exhibited significant global spatial clustering. Under complete spatial randomness, the observed mean nearest-neighbor distance (0.021) was substantially smaller than the expected distance (0.054), yielding a nearest-neighbor ratio of 0.39 and a z-score of −8.26 (p < 0.001). These findings indicate that EHDV-positive farms were aggregated into spatial clusters rather than randomly distributed across the study area. Local clustering of EHDV positivity was explored using Hot Spot Analysis (Getis-Ord Gi*) with a fixed distance band of 0.462 km, as determined by ArcGIS. Gi* z-scores for EHDV positivity ranged from −1.22 to 0.68 (all p ≥ 0.10), and no statistically significant hot or cold spots were detected, suggesting that EHDV-positive farms did not form distinct local clusters at this spatial scale despite the significant global clustering indicated by ANN. Kernel density mapping of EHDV-positive farms (cell size X km, bandwidth Y km) identified a pronounced band of increased case density extending along the coastal agricultural zones, with several overlapping high-intensity foci, while surrounding areas showed low or negligible density (Figure 1). These smoothed density patterns complement the ANN findings of significant global clustering, despite the absence of significant local Gi* hot spots at sub-kilometer scales.

### Univariate analysis of risk factors

The observed seroprevalence was higher (p = 0.416) in male camels (45.0%, CI 32.1–58.4) compared to females (39.4%, CI 34.3–44.7). Crossbred camels had a higher seroprevalence of 62.5% (CI 24.5–91.5) as compared to the local camels, which had a seroprevalence of 39.8% (CI 35.0–44.7), p = 0.195. The seroprevalence of EHDV differed significantly (p < 0.001) across the age categories, and the highest value was recorded in camels older than 4 years of age (45.9%, CI 40.4–51.4) followed by those below or equal to 4 years of age (19.3%, CI 11.7–29.1). Furthermore, the odds of testing positive were 3.4 times (p < 0.01) higher in older camels. A significantly (p = 0.002) higher seroprevalence of EHDV was recorded in the camels kept for leisure and breeding (43.1%, CI 37.9–48.4) purpose when compared with those kept for the race purpose (20.8%, CI 10.8–34.1). The seroprevalence was significantly (p = 0.007) higher in camels managed in close contact with other ruminants (49.3%, CI 40.7–57.9) as compared to those with no contact with these animals (35.6%, CI 30.0–41.6). The odds of testing positive were 1.8 times higher. The seroprevalence of EHDV was higher in camels infested with ticks (41.4%, CI 35.5–47.5) than in those without tick infestation (36.2%, CI 28.6–44.4), p = 0.341 (Table 2).

**Table 2:** Univariate analysis of epizootic hemorrhagic disease virus seroprevalence in camels from two governorates of Northern Oman.

Variable	Category	Pos. / Tested	Prevalence (%) (CI)	p-Value	OR (CI)
Governorate	Al Batinah North	135/329	41 (35.7–46.6)	χ²=0.415 p = 0.520	1.17 (0.72–1.92)
	Al Batinah South	32/86	37.2 (27–48.3)		Ref
Gender	Female	140/355	39.4 (34.3–44.7)	χ²=0.661 p = 0.416	Ref
	Male	27/60	45 (32.1–58.4)		1.26 (0.73–2.18)
Age Group	≤ 4 years	17/88	19.3 (11.7–29.1)	χ²=20.331 p < 0.001	Ref
	> 4 years	150/327	45.9 (40.4–51.4)		3.54 (1.99–6.27)
Breed	Local	162/407	39.8 (35–44.7)	χ²=1.681 p = 0.195	Ref
	Crossbred	5/8	62.5 (24.5–91.5)		2.52 (0.59–10.69)
Purpose	Breeding and Leisure	156/362	43.1 (37.9–48.4)	χ²=9.594 p = 0.002	2.89 (1.44–5.80)
	Race	11/53	20.8 (10.8–34.1)		Ref
Tick infestation	No	55/148	36.2 (28.6–44.4)	χ²=0.907 p = 0.341	Ref
	Yes	112/267	41.4 (35.5–47.5)		1.22 (0.81–1.85)
Contact	Camels only	98/275	35.6 (30–41.6)	χ²=7.187 p = 0.007	Ref
With other ruminants	69/140	49.3 (40.7–57.9)		1.76 (1.16–2.65)	

Variable = explanatory factor included in the model, Category = individual-level within each variable Positive / Tested: Number of positive animals divided by the total number examined in each category. Statistical significance: p < 0.05 considered statistically significant; p < 0.01 highly significant; p ≥ 0.05 not statistically significant. OR: Odds ratio with 95% confidence interval; OR > 1 indicates higher odds of positivity compared with the reference (Ref.) category, OR < 1 indicates lower odds; 95% CI not including 1.0 indicates a statistically significant association at the 5% level. OR = Odds ratio, CI = Confidence interval.

### Multivariate analysis

After univariate analysis, a multicollinearity analysis was conducted to check if the independent variables were highly correlated. All predictors had tolerance values above 0.1 and VIF values below 5, indicating that the model had no multicollinearity issues. All variables with a p-value < 0.25 (age, breed, camel purpose, and herd structure) were included in the initial binary logistic regression model. The model was refined using a backward stepwise exclusion method until only two significant predictors, namely, age and contact with other ruminants, remained. The final model indicated that camels above 4 years of age (OR 3.4) and those having contact with other ruminants (OR 1.6) were more likely to be EHDV-positive (Table 3). The Hosmer-Lemeshow Test (χ² = 0.355, p = 0.837) indicated that the model was a good fit to the data. However, its ability to explain the variability in EHDV seroprevalence was limited (Nagelkerke R² = 0.084).

**Table 3:** Binary logistic regression analysis of factors associated with epizootic hemorrhagic disease virus seropositivity in camels (n = 415) from two northern governorates of Oman.

Variable	Exposure Variable	Comparison	OR	CI	p-value
Age	> 4 years	≤ 4 years	3.4	1.88–5.96	< 0.01
Contact	With other ruminants	No contact	1.6	1.05–2.44	0.029

Variable = Factor included in the logistic regression model, Exposure variable = specific category of that factor being evaluated for association with EHDV seropositivity. Comparison: The reference category against which the exposure variable is compared when calculating the odds ratio OR: Odds ratio (OR) estimated from the binary logistic regression model CI: 95% confidence interval for odds ratio p-value: p-value from the Wald test in the logistic regression model; values < 0.05 were considered statistically significant. OR = Odds ratio, CI = Confidence interval.

## DISCUSSION

### Seroprevalence of EHDV in dromedary camels

This large-scale study investigated the seroprevalence of antibodies against EHDV in camels from two major governorates of Oman and attempted to identify potential risk factors associated with seropositivity using multivariate analysis. The seroprevalence observed in our study (40.2%) is comparable to that of a previous study in cattle from Oman, where it was recorded as 53.1% [23]. The TP estimate was higher than the apparent prevalence based on the reported sensitivity and specificity. There was little impact of false-positive results. Most of the remaining uncertainty in the adjusted estimates is due to the lack of precision in the sensitivity of the test. Although the presence of antibodies against EHDV was reported in camels from UAE a decade earlier [20], this is the first large-scale study undertaken in camels from Oman. EHDV antibodies were detected in camels from the UAE (29%), Pakistan (50%) and Sudan (17%) [20], and Mauritania (73%) [22]. Contrary to these findings, the overall apparent seroprevalence of EHDV in camels in Punjab, Pakistan, was 6.5%, with a TP of 7.3% [21]. This study documented substantial EHDV circulation along arid–humid coastal zones of Oman, in contrast to studies from Morocco [28] and Tunisia [29], which reported no evidence of infection in camels, suggesting marked regional heterogeneity in EHDV ecology across Afro-Eurasia. These observed differences could be attributed to differences in geography, vegetation cover, environmental factors, and herd structure. The climatic conditions in the sampled governorates are conducive for the vectors, and camels are usually managed in mixed herds with other ruminants, highlighting their potential use as sentinel animals for EHDV surveillance [28].

### Spatial patterns of EHDV seropositivity

Collectively, the spatial analyses indicate that EHDV-positive farms were not randomly distributed but exhibited clear aggregation at broader spatial scales. The strong global clustering detected by the average nearest-neighbor analysis, with a nearest-neighbor ratio of 0.39 and a highly significant z-score of −8.26, confirmed that under complete spatial randomness, infected farms tend to occur closer together than expected. The kernel density maps further strengthened these findings by revealing a broad band of elevated EHDV case density along the northern governorates, characterized by several overlapping foci of higher intensity embedded within generally low-density areas. These results indicated that EHDV transmission was structured into wider-area clusters or corridors of elevated risk rather than sharply defined local hotspots, which is consistent with a vector-borne disease influenced by landscape-level environmental and management factors rather than very fine-scale farm-to-farm spread [30]. During the sampling period (September 2020–July 2021), conditions would have included very high temperatures from late spring to midsummer and relatively milder, occasionally wetter conditions in winter and early spring, which are known to favor *Culicoides* breeding in irrigated and wadi-associated habitats [31–34]. Although no study was conducted in Oman to estimate the abundance and seasonal activity of *Culicoides*, in similar Gulf environments, *Culicoides* populations typically show a major activity peak in spring and a smaller peak in autumn when temperatures range roughly 20°C–30°C and relative humidity is moderate to high, with marked declines during the hottest months of June–July when mean temperatures exceed about 33°C–35°C [35].

### Risk factors associated with EHDV seropositivity

The prevalence recorded in both governorates was not significantly different, indicating that the exposure of camels to the virus is not influenced by their location. Both Al Batinah North and South have similar climatic and geographical features, and the camels are maintained under almost identical management conditions. EHDV is a vector-borne disease transmitted by midges (*Culicoides* spp.) [36], the results indicated that camels from these governorates were equally exposed to transmission. Furthermore, the higher temperatures and humidity in these coastal governorates might have facilitated transmission by reducing the extrinsic incubation period of the viruses within the midges [2, 9]. Proximity to large ruminants, higher environmental temperature and humidity, and fecal material accumulation result in elevated *Culicoides* activity [8, 37]. Regional climate models indicate that Oman’s average temperature has increased by 0.27 °C per decade and may rise another 2°C–4°C by the end of the century under high emissions, with less frequent but heavier rainfalls [38]. Such changes are expected to extend the duration of the hot season and increase the occurrence of extreme heat and drought. These conditions support the survival and biting activity of *Culicoides*, whereas intermittent heavy rainfall and cyclones create localized humid breeding sites near livestock farms. Thus, studying the disease in climatically and geographically diverse regions of Oman will be of greater interest to understand its epidemiology and contemplate a national control strategy.

Six variables (location, gender, age, breed, camel purpose, and herd structure) were evaluated for the seroprevalence of EHDV antibodies. Age, purpose of a camel, and structure of a herd (managed with other ruminants or alone) were found to be significantly (p < 0.05) associated in the univariate analysis. However, only age and contact with other ruminants were associated with the seroprevalence of EHDV in our sampled population. The final logistic regression model showed a modest explanatory power (Nagelkerke R² = 0.084), indicating that the included variables accounted for only a small proportion of the variability in EHDV seropositivity at the individual-level. This could be due to missing unmeasured factors such as ectoparasite intensity and vector control, *Culicoides* abundance, herd size and density, grazing patterns, and microclimatic conditions. More detailed data on management, ecology, and entomology are needed to improve predictive models of EHDV exposure in camels and clarify its drivers. Although the univariate analysis showed that camels kept for non-racing purposes were 2.89 times more likely to test positive, this variable was knocked out in the multivariate analysis, indicating that it could be a confounder.

Antibodies against EHDV increased with age, and camels aged > 4 years were more likely to test positive (OR, 3.4). A mixed finding was reported from camels from Pakistan, where EHDV antibodies were more prevalent among camels aged 3.1–7 years (9.3%) compared with those aged up to 3 years (4.8%) or above 7 years (2.9%) [21]. In the UAE, no EHDV antibodies were detected in younger racing dromedaries, whereas 29% of older breeding camels were positive [20]. Contrary to our results, studies in cattle [5, 39] and white-tailed deer [8] have shown that younger animals have a higher incidence. However, these studies focused on detecting viral RNA in the samples, whereas we detected antibodies against EHDV in the serum samples. EHDV infections in cattle are often subclinical or mild [2]. The camels sampled in our study might have been exposed to EHDV at an early age and later developed antibodies that persisted for years. Furthermore, older camels may have experienced multiple seasons of vector activity, leading to an increased likelihood of EHDV infection.

The results indicated that camels kept under mixed-species herd management were more likely (OR 1.6) to test positive. The prevalence of EHDV in mixed-species herds is notably influenced by the ecological interactions among various susceptible hosts and the local vector populations. A similar finding was reported in camels from Pakistan, where EHDV and BTV seroprevalence were found to be higher in animals managed under a mixed-animal farming system [21]. Furthermore, the high prevalence of EHDV antibodies in camels, such as 73% in Nouakchott, Mauritania, suggests that camels may experience subclinical infections and could serve as sentinel animals in endemic areas, particularly in contexts involving mixed livestock [22]. This finding implies potential cross-species transmission or shared exposure from other susceptible domestic hosts, such as sheep, goats, cattle, or buffalo. The general practice of rearing camels in mixed farming systems can increase their susceptibility to circulating infections among other domestic livestock species [21]. Collectively, the intermingling of diverse ruminant species in mixed herds or facilities can heighten the risk of EHDV exposure and propagation, with certain species potentially acting as amplifying hosts or providing valuable indicators for the disease’s presence and spread [2]. However, future research should include samples from all susceptible species in mixed herds and compare the seroprevalence to find the amplifying hosts of EHDV.

### Study limitations and future directions

The findings of this cross-sectional study should be interpreted with caution because they are limited to two governorates and may not be generalizable to the wider camel population in Oman. In addition, convenience sampling and owner willingness to participate may have introduced selection bias at both the herd and animal levels. No design effect was applied because the analyses focused on individual-level seroprevalence. The vector abundance and species composition, as well as the temporal patterns in *Culicoides* activity, were not measured. Therefore, we cannot link seropositivity to vector data or seasonal dynamics. Additionally, the study did not identify specific EHDV serotypes or perform molecular confirmation, making it impossible to determine which strains are currently circulating or whether active infection is ongoing. Despite these limitations, this study provides valuable baseline data and highlights the need for conducting longitudinal, multi-species, and entomological research in the future. Integrating climatic, seasonal, and molecular information will be essential to enhance EHDV risk assessment and guide effective control strategies in Oman.

## CONCLUSION

This first large-scale serological investigation confirms active circulation of EHDV among dromedary camels in Al Batinah North and South governorates of northern Oman, with an apparent individual-level seroprevalence of 40.2% (95% CI: 35.5–45.1%) and an estimated TP of 46.7% (95% CI: 41.3–52.3%). No significant difference in seroprevalence was observed between the two governorates (p = 0.520), reflecting similar climatic, geographical, and management conditions conducive to vector activity. Spatial analyses revealed significant global clustering of EHDV-positive holdings (nearest-neighbor ratio = 0.39, z = −8.26, p < 0.001), with elevated density along coastal agricultural corridors, although no local hot or cold spots were detected at sub-kilometer scales. Univariate analysis identified higher seropositivity in camels >4 years of age (45.9% vs. 19.3%, p < 0.001), those used for leisure/breeding (43.1% vs. 20.8%, p = 0.002), and those in contact with other ruminants (49.3% vs. 35.6%, p = 0.007). After multivariate adjustment, only age >4 years (OR = 3.4, 95% CI: 1.88–5.96, p < 0.01) and contact with other ruminants (OR = 1.6, 95% CI: 1.05–2.44, p = 0.029) remained independent risk factors.

These findings highlight the important role of dromedary camels in the multi-host ecology of EHDV in Oman, where mixed-species herding practices and favorable coastal conditions for *Culicoides* vectors likely facilitate transmission. The results underscore the need for integrated national surveillance programs that include camels as potential sentinel species, alongside cattle, sheep, and goats, to monitor EHDV circulation and prevent potential spillover or emergence in livestock systems. Practical implications include recommending vector control measures (e.g., manure management, insecticide use in high-risk coastal zones) and targeted awareness for farmers managing mixed herds, particularly those with older breeding animals.

Strengths of this study include its relatively large sample size (n = 415 camels from 50 holdings), use of a validated competitive ELISA with high specificity, rigorous adjustment for test imperfection via the Rogan–Gladen estimator, comprehensive univariate and multivariate risk factor analysis, and application of spatial statistics to reveal clustering patterns. The cross-sectional design, conducted across two epidemiologically similar governorates over nearly one year, provides robust baseline data in a previously understudied host species in Oman.

Limitations include the convenience sampling approach, which may introduce selection bias; restriction to northern coastal governorates, limiting generalizability to inland or southern regions; absence of vector abundance data, entomological surveys, or molecular serotyping to identify circulating strains; and reliance on serological evidence without confirmation of active viremia or clinical impact in camels. The modest explanatory power of the final logistic model (Nagelkerke R² = 0.084) suggests unmeasured factors (e.g., herd density, grazing patterns, microclimate) influence exposure.

Future research should prioritize longitudinal studies to assess seasonal dynamics and incidence, multi-species sampling in mixed herds to clarify amplifying hosts and cross-transmission risks, entomological investigations of Culicoides abundance and EHDV infection rates, and molecular characterization of circulating serotypes to inform vaccine or control strategies. In the context of projected climate warming in Oman, which may expand vector habitats and prolong transmission seasons, such integrated approaches will be essential for evidence-based disease management in changing environments.

In conclusion, this study establishes EHDV as an actively circulating pathogen in Omani camels and identifies key epidemiological features that support enhanced surveillance and risk mitigation in multi-host livestock systems. Addressing the identified gaps through collaborative, multi-disciplinary research will strengthen preparedness against orbiviral diseases in the region.

## DATA AVAILABILITY

All the generated data are included in the manuscript.

## AUTHORS’ CONTRIBUTIONS

MHH: Conceptualization. MHH, EIE, and HA: Methodology. MHH and EIE: Software. AA, MA, and AH: Validation. MHH and KA: Formal analysis. MHH, KA, MA, AA, and AH: Investigation. MHH and EIE: Data curation. MHH: Writing-original draft preparation. HA, KA, and MHH: Writing-review and editing. MHH: Visualization. All authors have read and approved the final version of the manuscript.
